# The Problem of Benzodiazepine Use and Its Extent in the Driver Population: A Population-Based Registry Study

**DOI:** 10.3389/fphar.2018.00408

**Published:** 2018-04-26

**Authors:** Francisco Herrera-Gómez, Eduardo Gutierrez-Abejón, Paloma Criado-Espegel, F. Javier Álvarez

**Affiliations:** ^1^Pharmacology and Therapeutics, Faculty of Medicine, University of Valladolid, Valladolid, Spain; ^2^Nephrology, Hospital Virgen de la Concha - Sanidad de Castilla y León, Zamora, Spain; ^3^Technical Direction of Pharmaceutical Assistance, Gerencia Regional de Salud de Castilla y León, Valladolid, Spain; ^4^CEIm Área de Salud Valladolid Este, Hospital Clínico Universitario de Valladolid - Sanidad de Castilla y León, Valladolid, Spain

**Keywords:** accidents, traffic, automobile driving, drug prescriptions, drug utilization, driving impairing medicines

## Abstract

**Background:** Benzodiazepines are driving-impairing medicines (DIM). This study presents current consumption of dispensed benzodiazepines in the Spanish general population, with a focus in pattern of use and concomitant medicines consumed with.

**Methods:** A population-based registry study was carried out to assess the year-2016 granted benzodiazepines dispensation in Castile and León. Weighting was performed to obtain the adjusted benzodiazepine consumption for licensed drivers according to age and gender using our national drivers' license census data.

**Results:** Benzodiazepines were used by 15.38% of the general population and 10.97% of drivers. Nearly 2% of the population and more than 1% of drivers took these medicines every day. The amount consumed (until 3 or more benzodiazepines *per* day) and concomitant use of other DIM were also higher. Women were the most frequent consumers, and anxiolytic use was usual. Consumption increases with age, but there were differences between men and women drivers from 60 years old.

**Conclusions:** The current use of benzodiazepines must serve to awareness of the healthcare personnel, patients, and authorities on their risks, above all on the road safety.

## Introduction

Road accident injuries, and death and disability that can cause, are important concerns for authorities, healthcare personnel, and drug developers. Promoting good practice related to addressing key behavior risk factors such drink and driving, the use of motorcycle helmets, or seat-belts and child restraints, are being done worldwide (WHO, 2015). In addition, there is increasing awareness that implementation of appropriate measures to avoid driving-impairing medicines (DIM) use by drivers has an impact on road accident occurrence (Schulze et al., 2012; WHO, 2016; Ramaekers, 2017).

Benzodiazepines are typical and well-known DIM, and there is a demonstrated relation between such and road accidents (Barbone et al., 1998; Meesmann et al., 2011). These drugs belong to the DRUID (Driving under the Influence of Drugs, Alcohol, and Medicines) category III, which forces their commercialization in Europe provided of harmonized warning labels in the product characteristics summary and package insert (Schulze et al., 2012). Both anxiolytic and hypnotic use involve a greater risk of death after a car crash (Barbone et al., 1998; Orriols et al., 2011), regardless of other causes of death among those using these medications (Dodds, 2017). In addition, the combined use with other psychotropic drugs is alarming, especially in countries where substance abuse is frequent (Schulze et al., 2012; Fierro et al., 2017). Improving prescription could therefore be of interest for people taking other DIM, and deserve a greater attention those with an actual need of these medications (Herrera-Gómez et al., 2018).

In general terms and from the perspective of public health, it is indispensable to prevent consumption of benzodiazepines and other DIM while driving. Warning labels are a mean to better inform healthcare providers and patients on the inherent risks of such medications (Ravera et al., 2012; Pollini et al., 2017). Deterrence of drivers with mandatory roadside testing become also a promising intervention, although its goal is rather diminishing road accidents than limiting DIM use (Fischer et al., 2017). Importantly, a clear and comprehensive information of all actors involved in the control of DIM consumption (practitioners and other health care providers, authorities, and the public at large) is a real and urgent necessity.

Knowledge of DIM consumption and patterns of use would allow to identify target populations to whom direct future interventions. In 2012, our team published data on drivers tested at random, showing a proportion of benzodiazepine users of 1.6% among all who had a positive result (Gómez-Talegón et al., 2012). Figures may be disquieting considering that proportion of positives may be higher among impaired drivers, as shown by other European study (Bezemer et al., 2014). In addition, and probably this is the case of Spain, consumers among drivers may be higher than is expected (prevalence of use in Spain has increased in the last decade: 18.7% of the general population (Schulze et al., 2012; Agencia Española de Medicamentos y Productos Sanitarios, 2017a; Ministerio de Sanidad, Servicios Sociales e Igualdad^1^).

In accordance with the problem addressed, this study presents the year-2016 consumption of dispensed benzodiazepines and other DIM in the largest region of Spain (Junta de Castilla y León^2^; Instituto Nacional de Estadística^3^). Adjusted consumption for licensed drivers is also presented in order to know use pattern differences corresponding to these medicines (Gutierrez-Abejón et al., 2017).

## Methods

In accordance with the STROBE recommendations (von Elm et al., 2008), a population-based registry study was carried out to assess the year-2016 granted benzodiazepines dispensation in Castile and León. Benzodiazepines were considered as DIM because these were dispensed provided of the pictogram ‘medicines and driving’ in the product characteristics summary and package insert (Real Decreto, 1345/2007; Agencia Española de Medicamentos y Productos Sanitarios, 2017b).

The CONCYLIA database (CONCYLIA^4^) which includes information on all medicines dispensed to the population covered by our public health system, were assessed (Table S1 in Supplementary Material). However, medicines dispensed at hospitals, in private clinics, and those considered as “over the counter” medications, were not considered.

As previously made (Gutierrez-Abejón et al., 2017), weighting was performed to obtain the adjusted benzodiazepine consumption for licensed drivers according to age and gender using the Castile and León drivers' license census data up to December 2016 (Ministerio del Interior^5^). With data on medicine dispensation *per* person, an anonymized dataset describing age and gender of consumers, benzodiazepines and concomitant DIM dispensed, number of doses, and date of dispensation, was generated. Based on the Anatomical Therapeutic Chemical code (ATC), benzodiazepines are classified as anxiolytic (N05BA) or hypnotic (N05CD), and this distinction was taken into account for the analysis. Our local ethics committee (CEIC/CEIm Área de Salud Valladolid Este) approved the study protocol (Reference number PI 17-646).

The following variables were considered: (1) the year-2016 benzodiazepine consumption, (2) acute (1–7 days), sub-acute (8–29 days) and chronic use (≥30 days) of benzodiazepines during the year 2016, (3) the year-2016 daily use of benzodiazepines, and (4) concomitant use of other DIM with benzodiazepines during the year 2016. Ethics Review Board approval was obtained (Reference number PI 16–387, approved on 17 March 2016).

Values obtained are presented, either as percentages with their 95% confidence interval (95% CI) or as means accompanied by their standard deviations (SD). Differences between continuous variables were calculated using Student's *t*-test, and those between categorical variables using Pearson's chi-squared. The level of significance was set at *p* ≤ 0.05. All statistical calculations were made by using the Statistical Package for the Social Sciences (SPSS version 23.0.; SPSS Inc, Chicago, IL).

## Results

**Figure 1:**
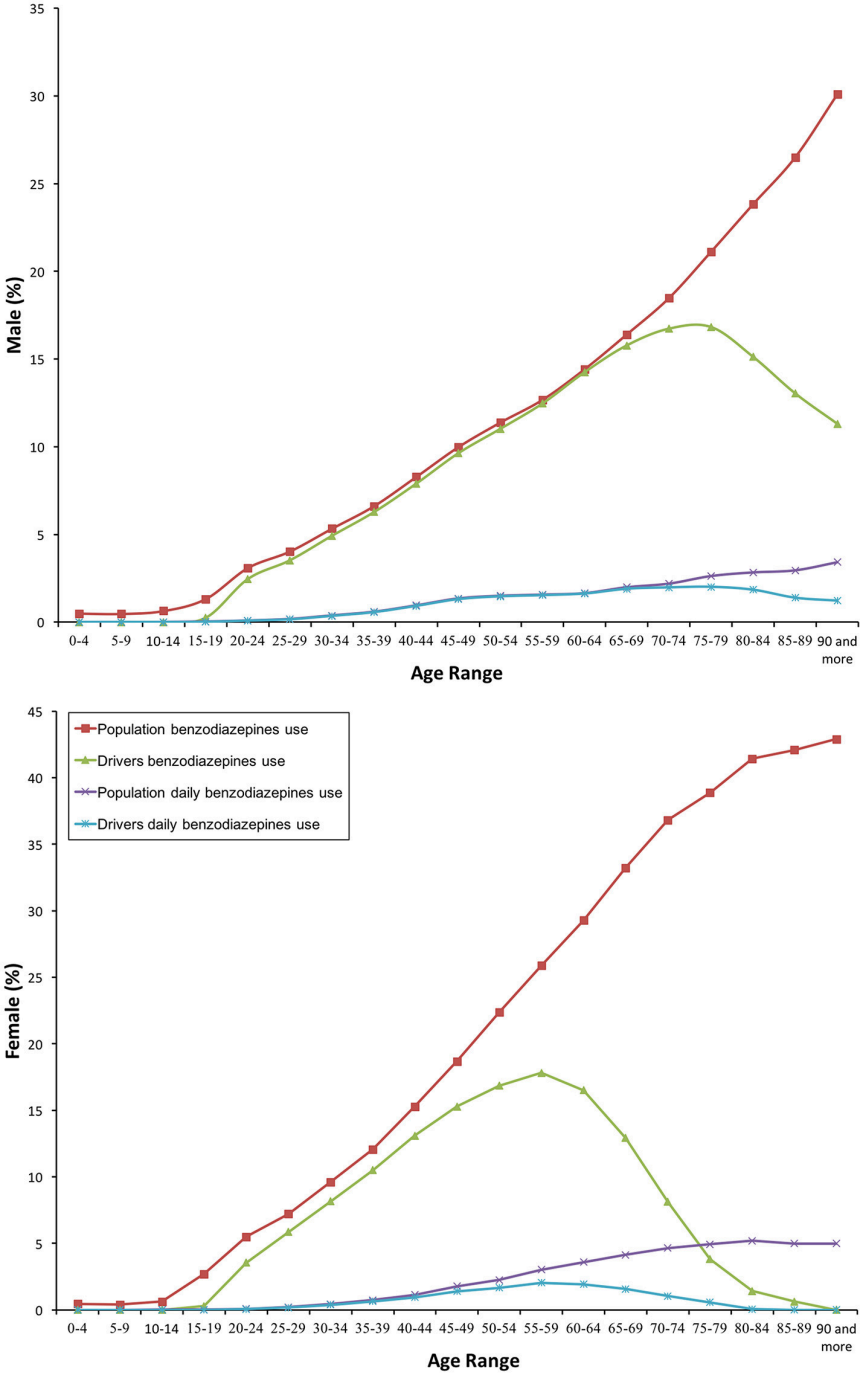
Frequency of consumption of benzodiazepines.

**Figure 2:**
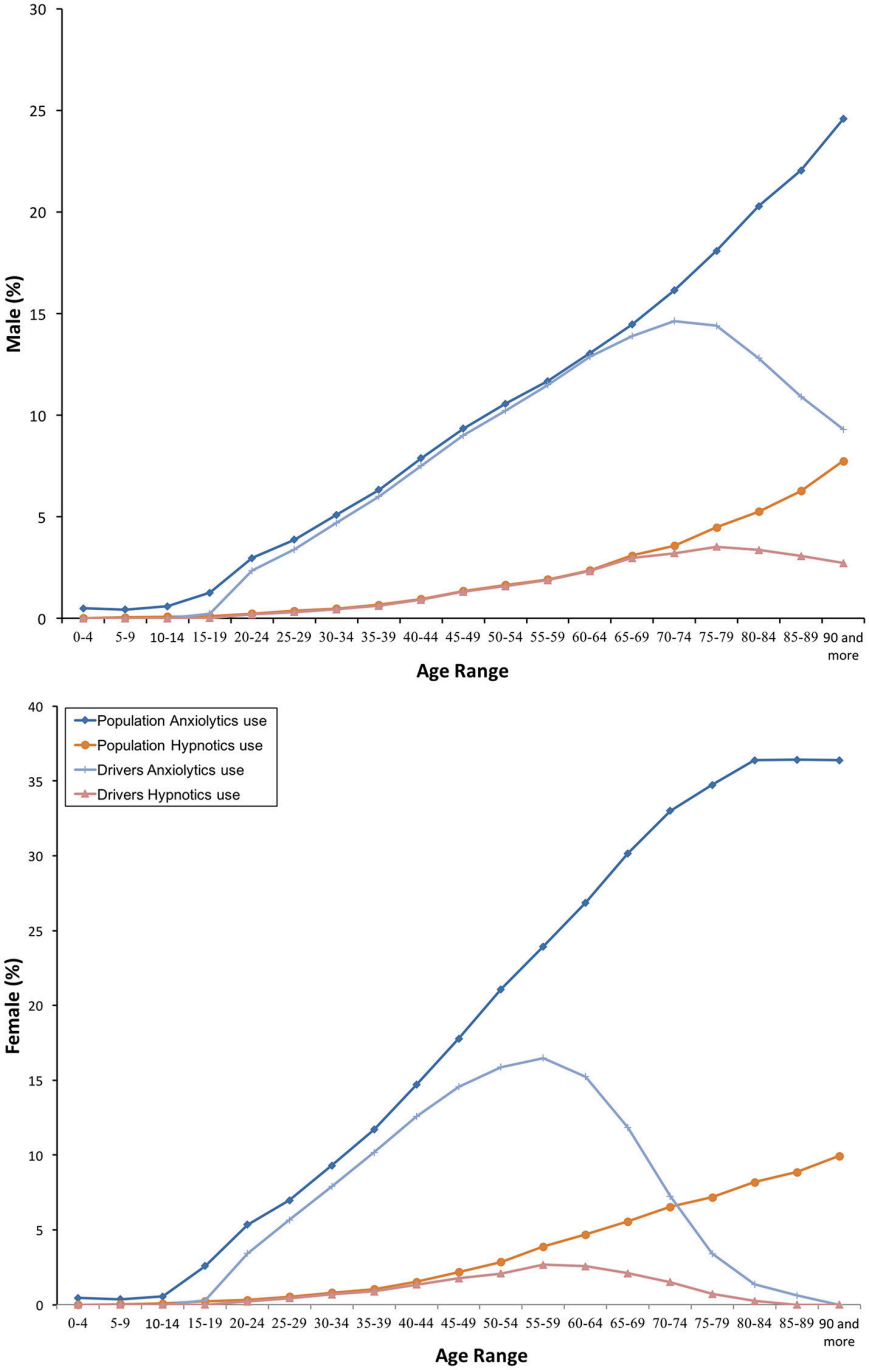
Frequency of consumption of anxyolitics and hypnotics benzodiazepines.

In 2016, benzodiazepines were dispensed to 15.38% of the general population. As shown in Table 1, chronic users were twice the acute and sub-acute users together (10.70 vs. 4.7%). Overall, anxiolytic benzodiazepines (N05BA, 13.98%) were more used than hypnotic benzodiazepines (N05CD, 2.48%). Daily use was of 1.69%, with an almost equal proportion of the anxiolytic and hypnotic use (0.9% versus 0.86%). On average, 3 or more molecules *per* day and 2 or more molecules *per* day were dispensed, respectively, to daily and non-daily users (Table 1). In all cases, consumers were more frequently females than males (Figure 1), and a trend toward increase in consumption of anxiolytic and hypnotic benzodiazepines may be observed as age increases (Figure 2).

**Table 1:** Benzodiazepine consumption according to CONCYLIA database and the Castile and León drivers' license census data.

	**General population using benzodiazepines % (95CI)**			**Drivers using benzodiazepines % (95CI)**		
	**Total**	**Anxiolytics**	**Hypnotics**	**Total**	**Anxiolytics**	**Hypnotics**
Total	15.38 (15.34–15.43)	13.98 (13.93–14.02)	2.48 (2.46–2.5)	10.97 (10.92–11.03)	10.16 (10.11–10.21)	1.55 (1.53–1.57)
Male	10.13 (10.07–10.18)	9.11 (9.05–9.16)	1.7 (1.68–1.73)	10.18 (10.12–10.24)	9.25 (9.19–9.31)	1.61 (1.58–1.63)
Female	20.47 (20.39–20.54)	18.69 (18.62–18.76)	3.24 (3.21–3.27)	12.17 (12.09–12.26)	11.52 (11.44–11.6)	1.45 (1.42–1.48)
**TYPE OF USE**						
**Chronic**						
Total	10.7 (10.66–10.74)	9.26 (9.22–9.3)	2.46 (2.44–2.48)	6.73 (6.69–6.77)	5.99 (5.95–6.03)	1.53 (1.51–1.55)
Male	6.83 (6.78–6.88)	5.74 (5.69–5.78)	1.68 (1.66–1.71)	6.66 (6.61–6.71)	5.68 (5.63–5.73)	1.59 (1.57–1.62)
Female	14.45 (14.38–14.51)	12.67 (12.61–12.73)	3.22 (3.19–3.25)	6.84 (6.77–6.9)	6.45 (6.39–6.51)	1.44 (1.41–1.47)
**Subacute**						
Total	4.18 (4.16–4.21)	4.26 (4.23–4.28)	0.02 (0.01–0.02)	3.73 (3.7–3.76)	3.82 (3.79–3.86)	0.01 (0.01–0.01)
Male	3.03 (3–3.06)	3.08 (3.05–3.11)	0.01 (0.01–0.02)	3.26 (3.23–3.3)	3.31 (3.27–3.35)	0.01 (0.01–0.02)
Female	5.3 (5.26–5.34)	5.39 (5.35–5.43)	0.02 (0.02–0.02)	4.44 (4.38–4.49)	4.6 (4.54–4.65)	0.01 (0.01–0.02)
**Acute**						
Total	0.49 (0.48–0.49)	0.46 (0.45–0.47)	0.01 (0–0.01)	0.36 (0.35–0.37)	0.34 (0.33–0.35)	0 (0–0)
Male	0.31 (0.3–0.32)	0.29 (0.28–0.3)	0.01 (0.01–0.01)	0.28 (0.27–0.29)	0.26 (0.25–0.27)	0 (0–0)
Female	0.66 (0.64–0.67)	0.63 (0.61–0.64)	0 (0–0.01)	0.48 (0.46–0.5)	0.47 (0.45–0.49)	0 (0–0)
**DAILY USE**						
Total	1.69 (1.67–1.7)	0.9 (0.89–0.91)	0.86 (0.85–0.87)	1.13 (1.12–1.15)	0.72 (0.7–0.73)	0.48 (0.47–0.49)
Male	1.15 (1.13–1.17)	0.64 (0.62–0.65)	0.57 (0.56–0.58)	1.18 (1.15–1.2)	0.72 (0.7–0.73)	0.52 (0.51–0.54)
Female	2.2 (2.17–2.23)	1.15 (1.13–1.17)	1.14 (1.12–1.16)	1.07 (1.04–1.09)	0.72 (0.7–0.74)	0.42 (0.4–0.43)
**AVERAGE OF DIM DISPENSED**						
**All benzodiazepines users**						
Total	2.25 (2.24–2.26)	2.25 (2.24–2.26)	2.65 (2.63–2.67)	2.13 (2.12–2.14)	2.12 (2.11–2.13)	2.71 (2.68–2.75)
Male	2.21 (2.2–2.22)	2.2 (2.19–2.21)	2.66 (2.63–2.7)	2.19 (2.18–2.2)	2.18 (2.17–2.2)	2.71 (2.67–2.76)
Female	2.27 (2.26–2.28)	2.27 (2.26–2.28)	2.65 (2.63–2.68)	2.06 (2.05–2.07)	2.06 (2.05–2.08)	2.69 (2.63–2.74)
**Daily benzodiazepines users**						
Total	2.96 (2.93–2.99)	3.23 (3.2–3.27)	2.76 (2.73–2.79)	3.06 (3.02–3.1)	3.25 (3.2–3.3)	2.9 (2.84–2.96)
Male	2.91 (2.88–2.95)	3.17 (3.11–3.23)	2.72 (2.66–2.77)	2.98 (2.94–3.03)	3.17 (3.11–3.23)	2.84 (2.77–2.92)
Female	2.99 (2.96–3.02)	3.26 (3.22–3.3)	2.78 (2.75–2.82)	3.18 (3.12–3.24)	3.37 (3.31–3.45)	3 (2.9–3.1)

*DIM, driving-impairing medicines*.

Similar findings come from the driver population: 10.97% used benzodiazepines and 1.13% took these medicines every day (Table 1). As in the general population, both anxiolytic and hypnotic benzodiazepines were used more frequently as age increased. However, female drivers decreased consumption over 60 years old, 15 years before male drivers (Figure 2).

Antidepressants were consumed concomitantly by somewhat more than half of daily benzodiazepine users (N06A, 55.82%). Almost one quarter used also opioids (N02A, 24.78%), other analgesics and antipyretics (N02B, 24.50%), or antipsychotics (N05A, 24.08%), and one fifth used anti-epileptics (N03A, 19.93%). Other DIM were also used, but less frequently (Table 2). There were no differences in concomitant DIM use between daily and non-daily users.

**Table 2:** Concomitant use of other DIM by daily benzodiazepine users in 2016.

**ATC code**	**Description**	**General population (%) 95CI**		**Drivers (%) 95CI**	
		**Male**	**Female**	**Male**	**Female**
**OTHER DIM CONSUMED BY DAILY BENZODIAZEPINE USERS IN 2016**					
N06A	Antidepressants	47.29 (46.45–48.13)	60.14 (59.55–60.73)	48.9 (47.94–49.86)	68.79 (67.64–69.94)
N02A	Opioids	17.48 (16.84–18.12)	28.48 (27.94–29.02)	16.29 (15.58–17)	23.54 (22.49–24.58)
N02B	Other analgesics and antipyretics	20.2 (19.52–20.88)	26.68 (26.15–27.21)	19.28 (18.52–20.04)	22.69 (21.66–23.73)
N05A	Antipsychotics	28.87 (28.11–29.64)	21.66 (21.16–22.15)	31.4 (30.51–32.29)	26.8 (25.7–27.89)
N03A	Antiepileptics	21.04 (20.35–21.72)	19.36 (18.89–19.84)	22.51 (21.7–23.31)	26.18 (25.09–27.27)
R06A	Antihistamincs for systemic use	7.89 (7.43–8.34)	8.1 (7.77–8.43)	7.04 (6.55–7.53)	7.07 (6.44–7.7)
A10B	Blood glucose lowering drugs, excl. Insulins	8.35 (7.88–8.82)	7.64 (7.32–7.96)	7.53 (7.02–8.04)	3.63 (3.17–4.09)
N05C	Hypnotics and Sedatives (exc. Benzodiazepines)	7.32 (6.88–7.76)	7.57 (7.25–7.89)	7.35 (6.84–7.85)	8.69 (8–9.39)
A10A	Insulins and analogs	4.95 (4.59–5.32)	4.28 (4.04–4.53)	4.86 (4.44–5.27)	2.32 (1.95–2.7)
N01B	Anesthetics, local	1.7 (1.48–1.92)	3.49 (3.27–3.71)	1.61 (1.36–1.85)	2.79 (2.38–3.19)
G04B	Urologicals	2.13 (1.88–2.37)	2.53 (2.34–2.72)	1.82 (1.56–2.07)	1.54 (1.24–1.85)
N06D	Anti-dementia drugs	1.76 (1.54–1.98)	2.5 (2.31–2.68)	1.08 (0.88–1.28)	0.16 (0.06–0.26)
N04B	Dopaminergic agents	2.09 (1.85–2.33)	2.07 (1.9–2.24)	1.48 (1.25–1.71)	0.68 (0.48–0.89)
N04A	Anticholinergic agents	3.49 (3.18–3.8)	1.32 (1.19–1.46)	4.11 (3.73–4.5)	2.02 (1.67–2.37)
N05B	Anxyolitics (exc. Benzodiazepines)	1.78 (1.56–2.01)	2.12 (1.95–2.29)	1.49 (1.26–1.72)	1.62 (1.31–1.94)
M03B	Muscle relaxants, centrally acting agents	1.61 (1.39–1.82)	1.97 (1.8–2.14)	1.76 (1.51–2.01)	2.64 (2.25–3.04)
A03B	Belladona and derivatives, plain	1.46 (1.25–1.66)	2.04 (1.87–2.21)	1.48 (1.25–1.71)	2.32 (1.95–2.7)
N02C	Antimigraine preparations	0.99 (0.82–1.16)	2.17 (1.99–2.34)	1.12 (0.92–1.33)	4.17 (3.68–4.67)

*ATC, Anatomical Therapeutic Chemical code; DIM, driving-impairing medicines*.

## Discussion

Our study shows that benzodiazepine use was frequent in both the general population and among drivers. Nearly 2% of the population and more than 1% of drivers took these medicines every day. The amount consumed and concomitant use of other DIM were also higher, being figures alarming among daily users. Women were the most frequent consumers, and anxiolytic benzodiazepines the most frequently used. Consumption increases with age, but there were differences between men and women drivers from 60 years old.

Our national data (Agencia Española de Medicamentos y Productos Sanitarios, 2017a; Ministerio de Sanidad, Servicios Sociales e Igualdad^1^) and the published literature on benzodiazepine consumption (Magrini et al., 1996; Jeantaud et al., 2001; Petitjean et al., 2007; Ramadan et al., 2016) confirm that these medicines are quite frequently used. Higher daily doses and concomitant use of other DIM are also consistent with available evidence (Haw and Stubbs, 2007; Maric et al., 2017). It is well know that the amount consumed and poly-substance use are related to an increased risk of being involved in a fatal road accident (Barbone et al., 1998; Orriols et al., 2011; Schulze et al., 2012; Rudisill et al., 2014).

Importantly, mental and behavioral disorders are important risk factors to become daily benzodiazepine user, for the need of higher doses, and concomitant use of other DIM (Maric et al., 2017). Accordingly, appropriate prescription of these medicines is very important. External pressures, customary behaviors and the feeling of greater knowledge about usual drugs than it is warranted (de las Cuevas and Sanz, 2004) emphasize that education of healthcare professionals is an objective to reach. Blanket medication orders, especially in managing multi-morbidity patients, is also to avoid (González López et al., 2016). In our opinion, benzodiazepines are not properly used because adjustment of doses during longer periods is challenging, considering tolerance (Magrini et al., 1996; Jeantaud et al., 2001; Petitjean et al., 2007; Ramadan et al., 2016).

The fact that women appear as the most common users of benzodiazepines is also consistent with available evidence. To be a woman constitutes a risk factor to the use of benzodiazepines, particularly if lower incomes prevent to opt for a better treatment (Cunningham et al., 2010). Probably, this is a hard problem in developing countries (Srisurapanont et al., 2005; Dièye et al., 2006), and it should not occur in countries where healthcare is free and universal as Spain, and where a large proportion of the cost of medicines dispensed is subsidized. Nevertheless, the amount of benzodiazepines consumed was higher, and it emphasizes once more that an adequate prescription is urgently needed.

Consumption of benzodiazepines increased with age, and a sharp increase may be observed from 60 years old. Although differences can be found depending on if general practitioners or specialists are the prescriptors (Mell et al., 2017), this global problem began more than 20 years (Magrini et al., 1996; Jeantaud et al., 2001; Petitjean et al., 2007; Ramadan et al., 2016). As age increases, people are more sensitive for a longer benzodiazepine therapy and for higher doses (Cheng et al., 2008), although disorders remain the same to those present in younger populations (Jacob et al., 2017). In addition, patients could also take more of the drug than directed without inform their treating physicians. Measures to control the amount prescribed are therefore needed.

Interestingly, there were differences in the use of benzodiazepines between men and women drivers from 60 years old. It seems that women drive less than men as they age. It should not be forgotten that benzodiazepines belong to the DRUID category III, and are marketed in our country and Europe provided of a harmonized and mandatory pictogram (Real Decreto, 1345/2007; Agencia Española de Medicamentos y Productos Sanitarios, 2017a). As a public health intervention, the pictogram “medicines and driving” serves as a vehicle to better inform healthcare providers about driving-related risks of these medications, and as a reminder to relay this information to patients (Ravera et al., 2012; Pollini et al., 2017). Nevertheless, as it has been intuitively stated, other interventions are needed (improving prescription, awareness of the population, etc.).

Remarkable, in the last years, the importance of patient and family involvement in Health Technology Assessment (HTA) is becoming widely recognized, as they are directly affected in decisions on what medicine and at what doses are needed for treating a given disease (EUPATI^6^). A patient well informed is therefore a key objective (European Medicines Agency^7^). With respect directly to the use of benzodiazepines and other psychoactive drugs, this could limit reckless self-medication.

As a response to the need for providing a more clear and comprehensive information to healthcare providers, patients and drivers, data presented should also be transmitted to authorities in order to improve interventions destined to correct benzodiazepine consumption. These interventions must be associated to current measures, such as deterrence of drivers in all modalities (mandatory on-road testing, informative roadside campaigns, etc.; Fischer et al., 2017).

Our study has limitations. Benzodiazepines dispensation cannot be considered as an equivalent to benzodiazepine consumption, as beneficiaries of our health system could having not consumed such medicines. Although a minority, both hospital-dispensed and over-the-counter medicines are not included in the data presented here. In our opinion, little changes are expected with the inclusion of this information. Our results are based on almost all benzodiazepines consumed, as our public health system dispensed more than 95% of the total amount consumed (Junta de Castilla y León; Instituto Nacional de Estadística^3^). Nevertheless, other psychoactive medications could be relevant in the context of minor surgery procedures, endoscopy, etc. when patients are discharged after completed such procedures. In addition, the effect of herbal products that may be consumed in combination to benzodiazepines is not considered. Herbal sedatives have been used all over the world to treat insomnia and anxiety for thousands of years. The problem of consumption of such products may be greater in developing countries (WHO, 2005), and highlights differences existing concerning the use of benzodiazepines and other psychoactive drugs between these countries and developed countries. Finally, the CONCYLIA database does not contain information on consumption of medicines by drivers, and weighting was performed to adjust the consumption of benzodiazepines among licensed drivers by age and gender, as had made in a previous study (Gutierrez-Abejón et al., 2017). Therefore, there may be subtle differences with respect to other studies.

## Conclusions

Our study shows that benzodiazepines are frequently used. Nearly 2% of the population and more than 1% of drivers took these medicines every day. The amount consumed and concomitant use of other DIM were also higher. Women were the most frequent consumers, and anxiolytic use was usual. Consumption increases with age, but there were differences between men and women drivers from 60 years old.

These findings that are consistent with available evidence, must serve to inform the healthcare personnel, above all those who may be unaware of the problem, as well as patients, their families, the population at large, and authorities on the current use of benzodiazepines.

## Author contributions

FJA developed the hypothesis and study design. EG-A and PC-E performed statistical analysis. FH-G and FJA drafted the manuscript. All authors have given final approval for this paper to be published.

### Conflict of interest statement

The authors declare that the research was conducted in the absence of any commercial or financial relationships that could be construed as a potential conflict of interest.

## Footnotes

^1^Ministerio de Sanidad, Servicios Sociales e Igualdad. Secretaria de Estado de Servicios Sociales e Igualdad. Delegacion del Gobierno para el Plan Nacional sobre Drogas. Encuesta sobre alcohol y drogas en Espana: EDADES 2015- 2016/[Survey on alcohol and drugs in Spain: AGES 2015-2016]. http://www.pnsd.msssi.gob.es/profesionales/sistemasInformacion/sistemaInformacion/pdf/2015_EDADES__Informe_pdf (Accessed 5 Nov 2017).

^2^Junta de Castilla y León. *IV Plan de Salud de Castilla y León: Perspectiva 2020/[IV Health Plan of Castilla y León: 2020 Perspective]*. Available online at: http://www.jcyl.es/junta/cp/Analisis_situacion_IVPdS.pdf (Accessed 5 November 5, 2017).

^3^Instituto Nacional de Estadística. *Cifras oficiales de población de los municipios españoles: Revisión del Padrón Municipal/[Official population figures of Spanish municipalities: Municipal Register Review]*. Available online at: http://www.ine.es/dyngs/INEbase/en/operacion.htm?c=Estadistica_C&cid=1254736177011&menu=resultados&idp=1254734710990 (Accessed November 5, 2017).

^4^CONCYLIA. *Sistema de Información de Farmacia*. Gerencia Regional de Salud de Castilla y León. Available online at: http://www.saludcastillayleon.es/portalmedicamento/es/indicadores-informes/concylia (Accessed November 5, 2017).

^5^Ministerio del Interior. Dirección General de Tráfico. *Estad*í*sticas e Indicadores. Permisos de conducción/[Driving licenses]*. Available online at: http://www.dgt.es/es/seguridad-vial/estadisticas-e-indicadores/permisos-conduccion/ (Accessed November 5, 2017).

^6^EUPATI. *Guidance for patient involvement in HTA*. Available online at: https://www.eupati.eu/health-technology-assessment/guidance-for-patient-involvement-in-hta/ (Accessed November 5, 2017)

^7^European Medicines Agency. *Revised framework for interaction between the European Medicines Agency and patients and consumers and their organizations. EMA/637573/2014*. Available online at: http://www.ema.europa.eu/docs/en_GB/document_library/Other/2009/12/WC500018013.pdf (Accessed November 5, 2017).
