# Supplementary material for: The Problem of Benzodiazepine Use and Its Extent in the Driver Population: A Population-Based Registry Study
Source: Front Pharmacol. 2018 Apr 26;9:408. doi: 10.3389/fphar.2018.00408 (PMC5933078; doi:10.3389/fphar.2018.00408)
Supplement: Supplementary file 1 [file Table_1.DOCX]

**Table S1.** Demographic characteristics of the population.

|  | **General population**^£^  up to December 2016 | | | **Licensed drivers**^$^  up to December 2016 | | |
| --- | --- | --- | --- | --- | --- | --- |
|  |  |  |  |  |  |  |
| **Total** | **2,371,008** | | | **1,471,983** | | |
| **Sex** |  |  |  |  |  |  |
| Males | 1,165,493 | | | 884,021 | | |
| Females | 1,205,515 | | | 587,962 | | |
| **Age Range** (Male/Female) |  |  |  |  |  |  |
| 0 to 4 | 44,382 | / | 41,386 | – | | |
| 5 to 9 | 50,665 | / | 47,821 | – | | |
| 10 to 14 | 49,847 | / | 47,730 | – | | |
| 15 to 19 | 48,862 | / | 46,935 | 9,238 | / | 5,634 |
| 20 to 24 | 53,230 | / | 52,333 | 42,165 | / | 34,280 |
| 25 to 29 | 61,109 | / | 59,382 | 53,617 | / | 48,755 |
| 30 to 34 | 71,742 | / | 68,841 | 66,192 | / | 58,489 |
| 35 to 39 | 87,267 | / | 83,676 | 83,112 | / | 72,880 |
| 40 to 44 | 92,967 | / | 90,094 | 88,673 | / | 76,856 |
| 45 to 49 | 93,035 | / | 91,392 | 89,641 | / | 74,389 |
| 50 to 54 | 93,251 | / | 91,395 | 90,290 | / | 69,036 |
| 55 to 59 | 88,988 | / | 85,894 | 87,607 | / | 59,781 |
| 60 to 64 | 75,073 | / | 72,029 | 74,219 | / | 40,173 |
| 65 to 69 | 66,403 | / | 67,268 | 63,824 | / | 26,068 |
| 70 to 74 | 58,076 | / | 63,396 | 52,595 | / | 13,755 |
| 75 to 79 | 43,540 | / | 53,807 | 34,852 | / | 5,277 |
| 80 to 84 | 44,319 | / | 62,772 | 28,194 | / | 2,055 |
| 85 to 89 | 28,618 | / | 47,555 | 14,133 | / | 484 |
| 90 or more | 14,119 | / | 31,809 | 5,669 | / | 50 |

^£^Source: CONCYLIA database. ^$^Source: the Castile and León drivers’ license census.
